# Supplementary material for: LEM4 confers tamoxifen resistance to breast cancer cells by activating cyclin D-CDK4/6-Rb and ERα pathway
Source: Nat Commun. 2018 Oct 9;9:4180. doi: 10.1038/s41467-018-06309-8 (PMC6177406; doi:10.1038/s41467-018-06309-8)
Supplement: Supplementary file 1 — Supplementary Information [file 41467_2018_6309_MOESM1_ESM.pdf]

Supplementary Information

LEM4 confers tamoxifen resistance to breast cancer cells by activating cyclin D-CDK4/6-Rb and ERα pathway

Gao *et al.*

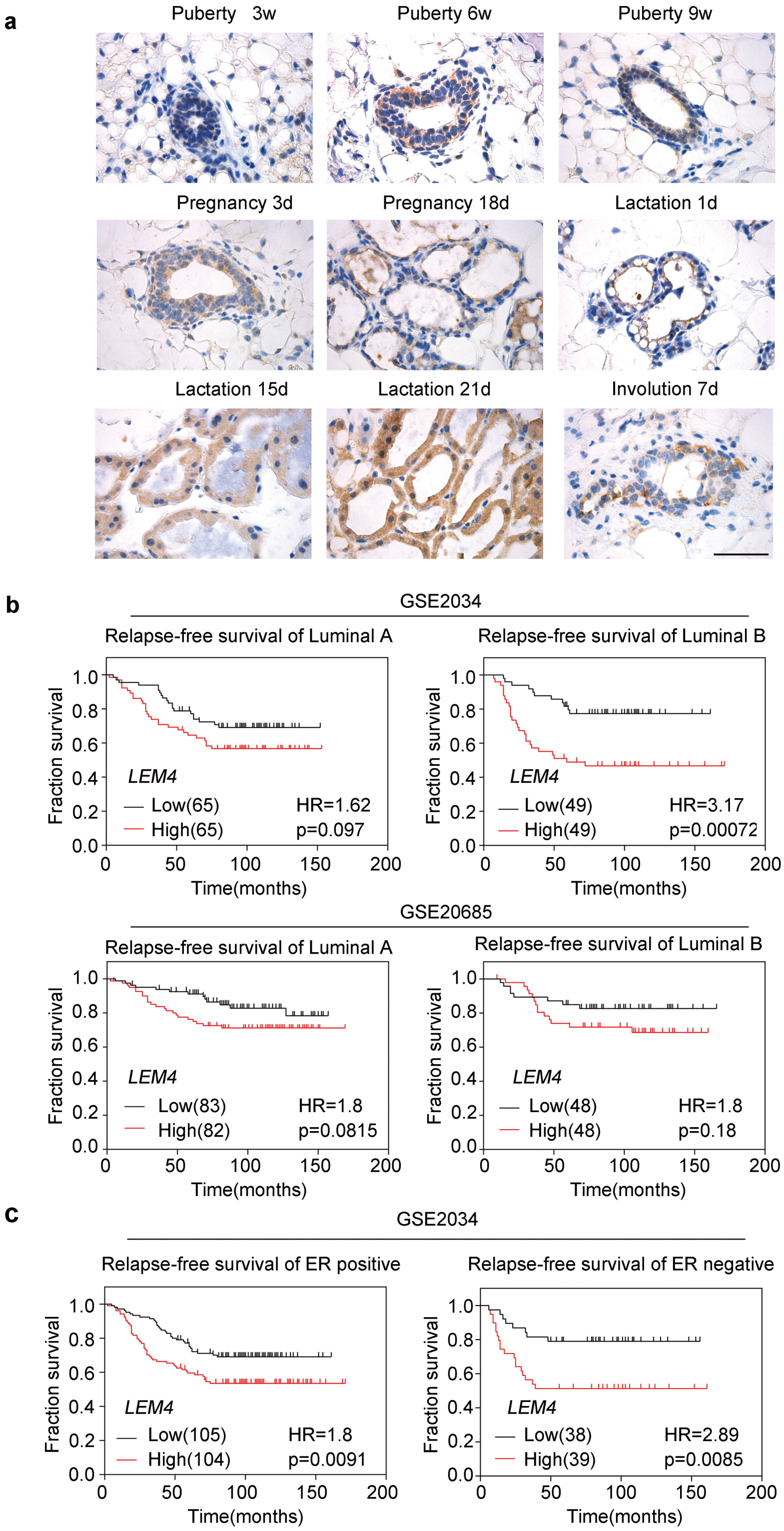

Supplementary Figure 1. High expression of *LEM4* correlates with poor survival in breast cancers. (a) IHC for Lem4 in mouse mammary glands during four different stages, puberty, pregnancy, lactation, and involution. Scale bar: 50 μm. **(b)** Kaplan–Meier analysis of relapse-survival in luminal A subtype and luminal B subtype breast cancers data from GEO datasets. Samples were separated into two groups using the median expression of LEM4 as the dividing line. *P*-values were calculated by the log–rank test. **(c)** Kaplan–Meier analysis with median cutoff values of LEM4 expression for samples stratified according to ERα status. *P*-values were calculated by the log–rank test.

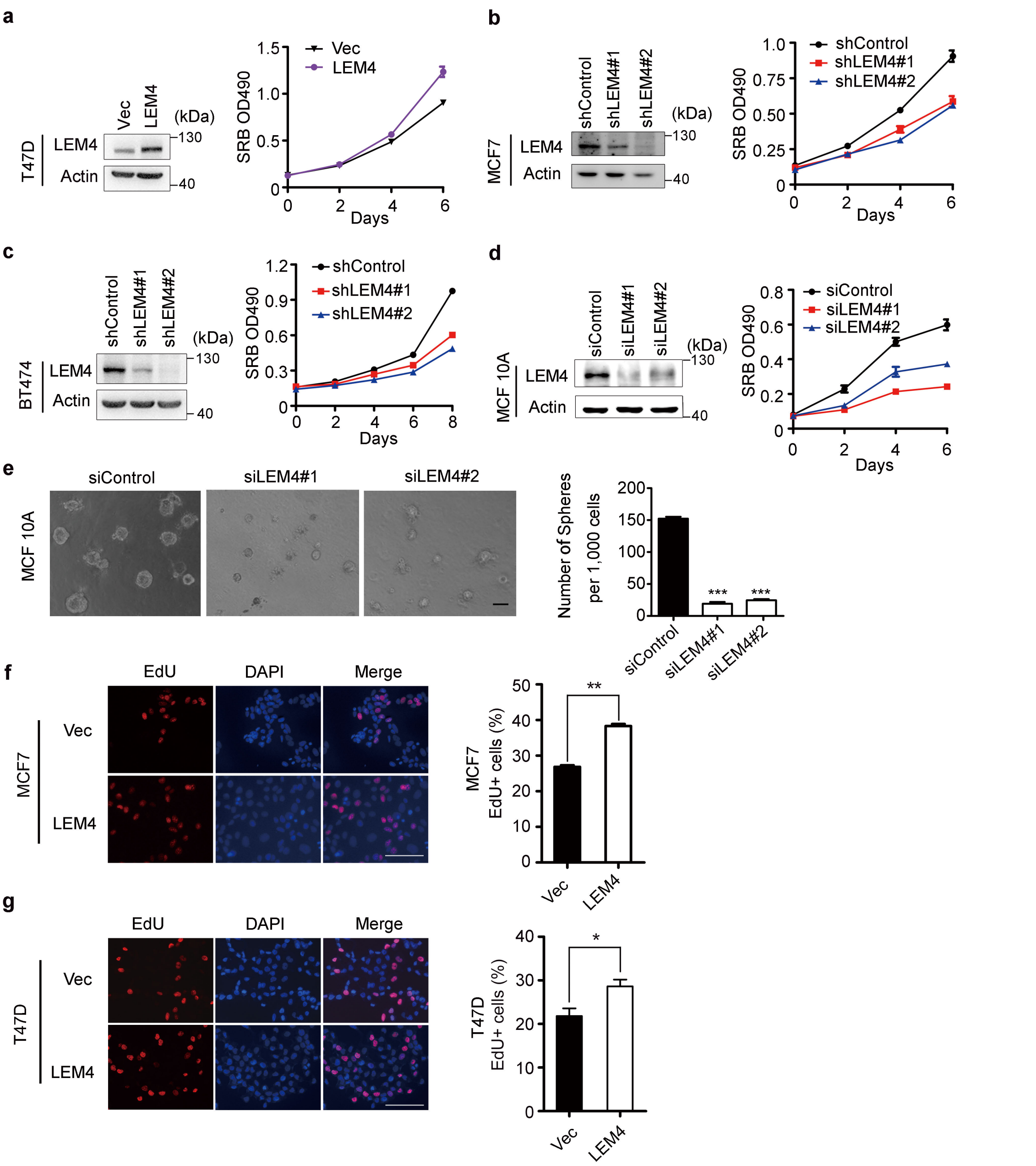

Supplementary Figure 2. Effect of LEM4 expression on the proliferation of breast cancer cells. (a) Immunoblot was performed with anti-LEM4 antibody in T47D-LEM4 cells. Growth curve were measured by SRB assay in monolayer culture. **(b)** Western blot was performed with anti-LEM4 antibody in LEM4-depleted MCF7 cells. Growth curve were measured by SRB assay in monolayer culture. **(c)** Growth curves of shControl-BT474, shLEM4#1-BT474, and shLEM4#2-BT474 cells grown in monolayer culture. **(d)** Growth curves of siControl or siLEM4 treated MCF-10A cells grown in monolayer culture. **(e)** Knockdown of LEM4 significantly reduces the number of spheres with a diameter greater than 75 mm. The data are presented as the number of defined mammospheres per 1,000 seeded cells. Representative bright field images for spheres were shown for each group. Scale bar: 100 μm. **(f and g)** EdU incorporation assay was performed in MCF7-vec and MCF7-LEM4 cells (f), and T47D-vec and T47D-LEM4 cells (g). Scale bar: 100 μm. Mean ±s.d. for three independent replicates. *P<0.05, **P<0.01, and ***P<0.001. Tukey's multiple comparisons test for e. Student's t-test for f and g.

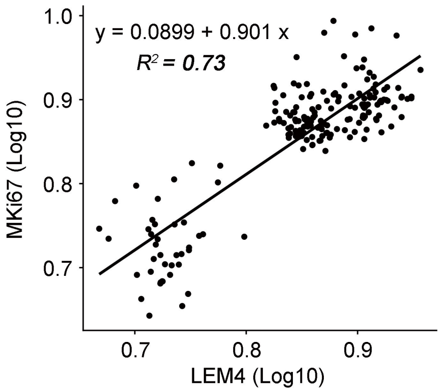

Supplementary Figure 3. Pearson's correlation between *LEM4* and *MKI67* mRNA levels in samples from GEO GSE2990.

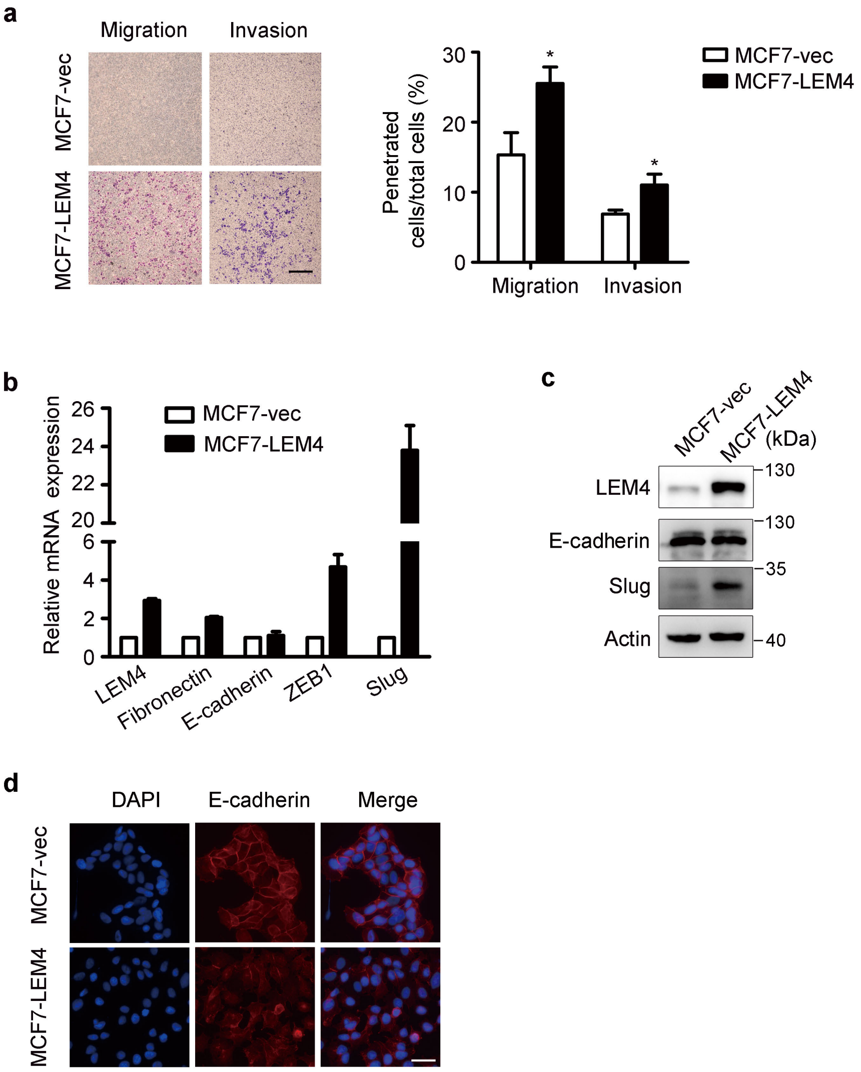

Supplementary Figure 4. LEM4 overexpression promotes MCF7 cells migration and invasion. (a) MCF7-vec and MCF7-LEM4 cells were subjected to migration and invasion assays. MCF7-LEM4 cells leads to significant increase of cell migration and invasion. Data represent Mean ± s.d., triplicates. Scale bar: 50 μm. **(b)** Real-time RT-PCR analysis of EMT markers in MCF7-LEM4 cells. **(c)** Immunoblots of Slug and E-cadherin in MCF7-LEM4 cells. **(d)** MCF7-LEM4 cells were immunostained with anti-E-Cadherin (red) antibody, and counterstained with DAPI (blue). Scale bar: 20 μm. **P*<0.05, Student's *t*-test for a.

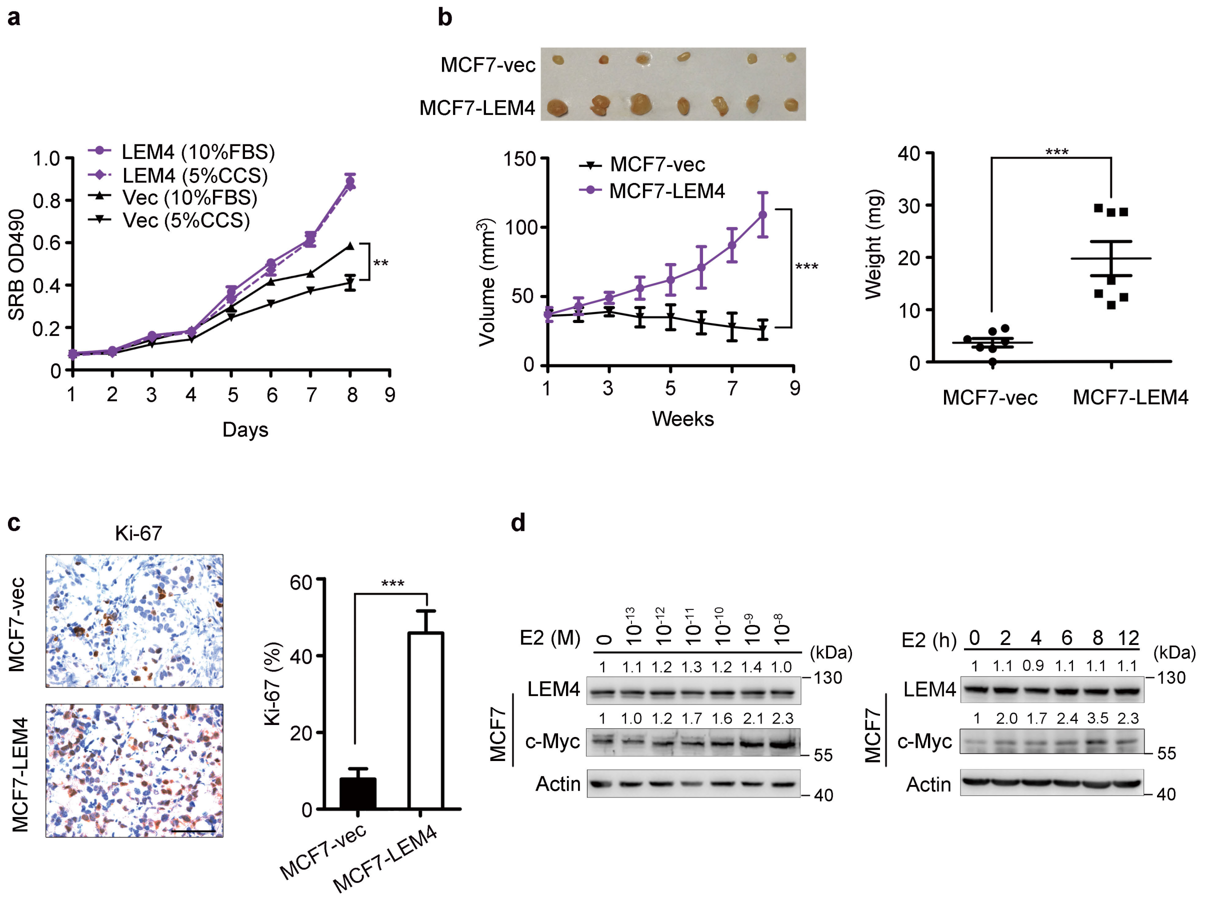

Supplementary Figure 5. LEM4 promotes MCF7 cell proliferation and tumorigenesis without estrogen. (a) Growth curves of MCF7-vec and MCF7-LEM4 cells grown under estrogen-deprived conditions (DMEM phenol-free medium containing 5% dextran-coated charcoal-stripped serum) or in DMEM supplemented with 10% FBS. **(b)** Tumor growth curves and tumor weight of MCF7-vec and MCF7-LEM4 cells implanted subcutaneously in athymic mice without estrogen pellet implantation. **(c)** Immunohistochemical analysis of ki-67 in MCF7-vec and MCF7-LEM4 tumor xenografts from (b). Mean ± s.d. for three independent replicates. **(d)** Immunoblot analysis of LEM4 and c-Myc expression in MCF7 cells treated with a time and dosage course of E2. ***P*<0.01, ****P*<0.001. Repeated measures ANOVA for a, b (volume). Student's *t*-test for b (weight) and c.

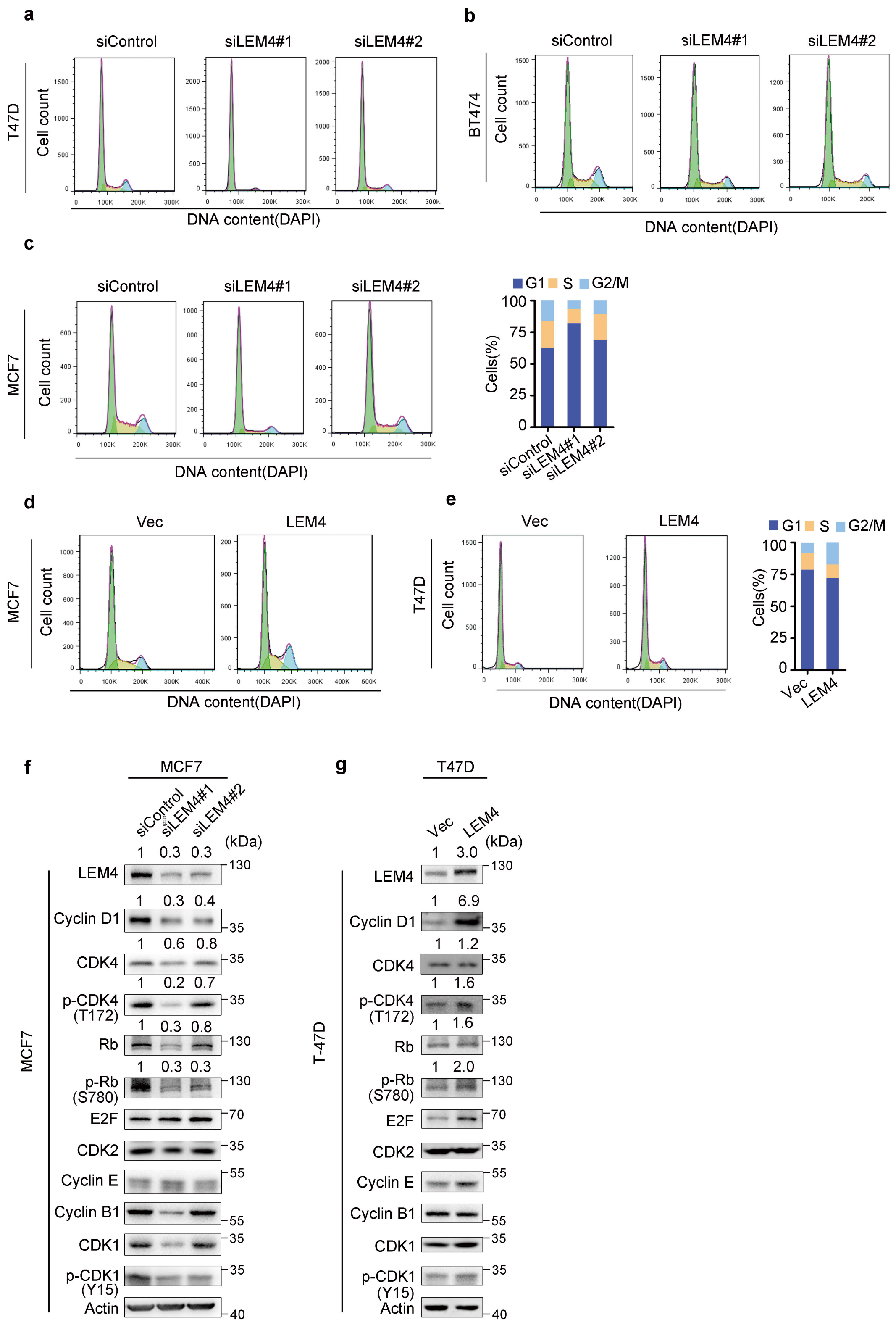

Supplementary Figure 6. LEM4 alters the cell cycle. (a-c) Representative results of FACS experiments with LEM4-depleted T47D (a), BT474 (b), and MCF7 (c) cells. **(d and e)** FACS analysis of the proportion of cells in the G1, S, and G2/M phases with MCF-LEM4 (d) and T47D-LEM4 cells (e). **(f and g)** Immunoblot of cell cycle-related gene expression using the indicated antibodies in LEM4-depleted MCF7 cells (f) and T47D-LEM4 cells (g).

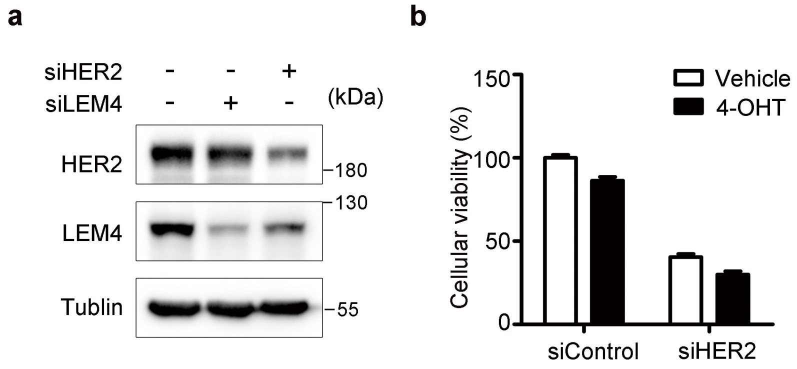

Supplementary Figure 7. Knockdown of HER2 reduces LEM4 expression and reverses tamoxifen resistance in BT474 cells. (a) Immunoblot analysis of LEM4 and HER2 expression in BT474 cells treated with LEM4-si and HER2-si for 48 hours respectively. **(b)** HER2 siRNA- and control siRNA-treated BT474 cells were treated with and without tamoxifen (1.0 μM) for 6 days. Total cell viability was assessed by SRB assays. Mean ± s.d. for three independent replicates.

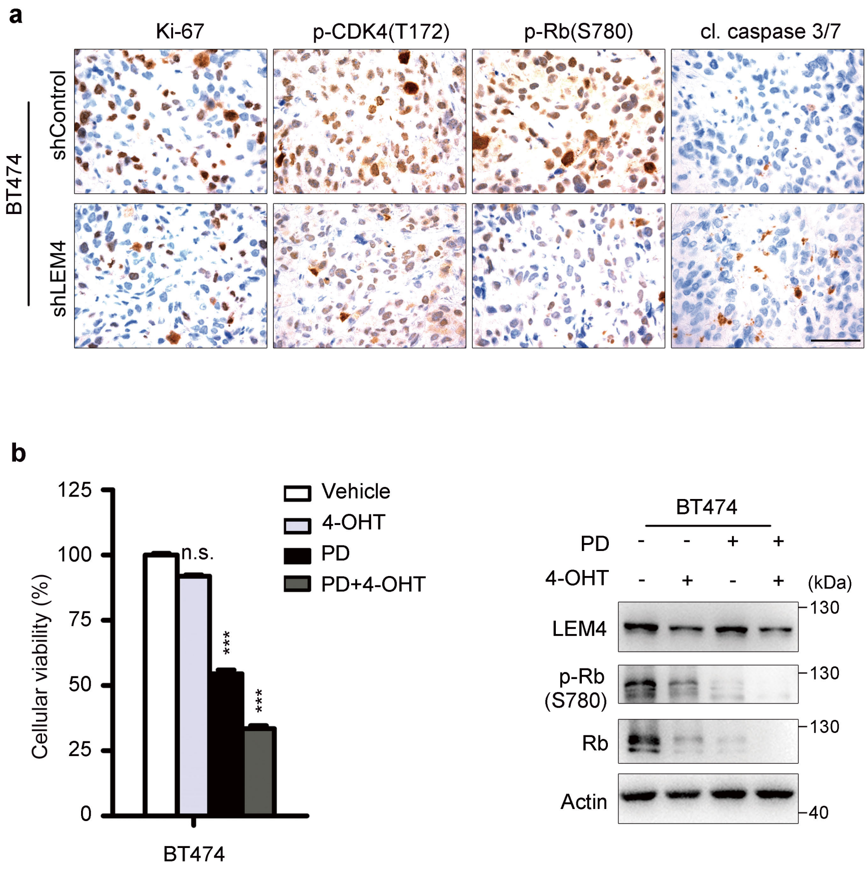

Supplementary Figure 8. Reduction of the phosphorylation levels of CDK4 and Rb in BT474 cells reverses tamoxifen resistance. (a) IHC for Ki-67, p-CDK4, p-Rb, and cleaved caspase 3/7 of tumors. (Tumor growth of BT474-shcontrol and BT474-shLEM4 cells as subcutaneous xenografts in athymic mice with E2 supplementation until tumors reached ~200 mm^3, then treated with tamoxifen pellets implanted subcutaneously.) Scale bar: 50 μm. **(b)** BT474 cells were treated with 5% DCC-FBS (control), 4-OHT (1 μM), PD0332991 (PD) (0.2 μM), or a combination of 4-OHT and PD0332991. Adherent cells were tested by SRB after 9 days. Immunoblots for Rb and p-Rb of the treated cell lysates. Data are presented as % parental control. Mean ± s.d. for three independent replicates. n.s., not significant, ***P<0.001. Student's t-test for b.

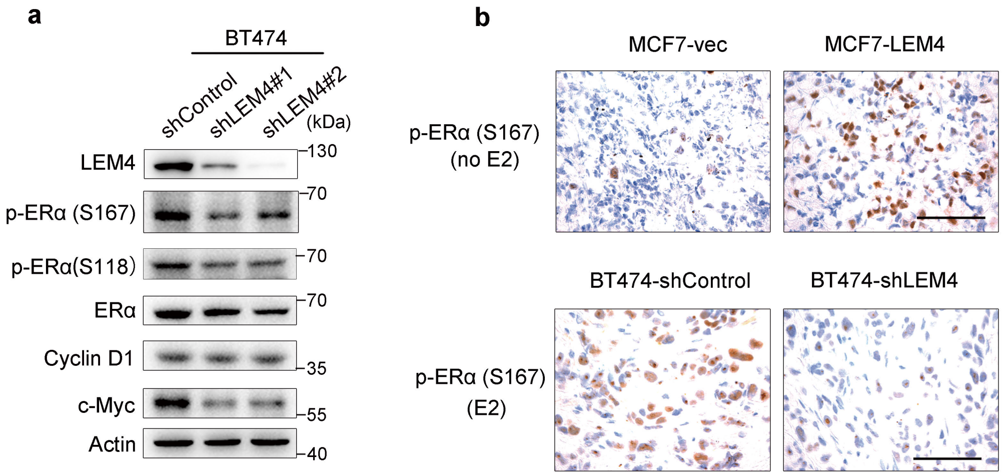

Supplementary Figure 9. LEM4 regulated the phosphorylation levels of ERα-Ser167. (a) LEM4-depleted BT474 cells were performed immunoblot assay with indicated antibodies. **(b)** IHC for p-ERα-Ser167 in MCF7 (a) or BT474 (b) cells as subcutaneous xenografts in athymic mice with or without E2 pellet. Scale bar: 50 μm.

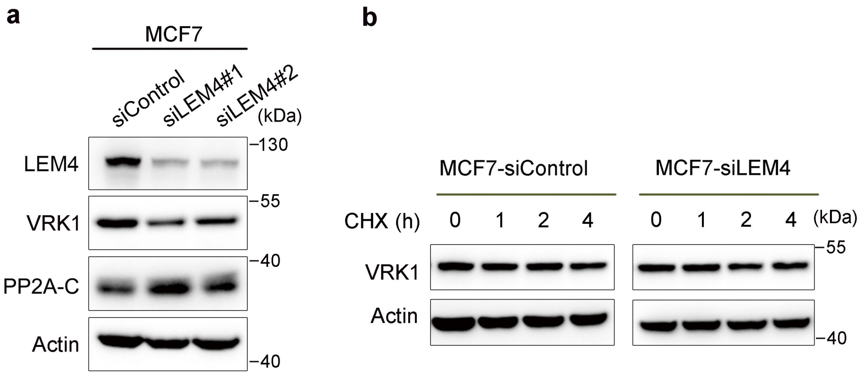

Supplementary Figure 10. VRK1 protein degradation is independent on LEM4. (a) MCF7 cells was transfected with LEM4 siRNA. After 48 hours of incubation, immunoblot was performed with indicated antibodies. **(b)** MCF7-shcontrol and MCF7-shLEM4 cells were treated with 50 μg mL^{-1} CHX for 0, 1, 2 and 4 hours and VRK1 analyzed by immunoblot.

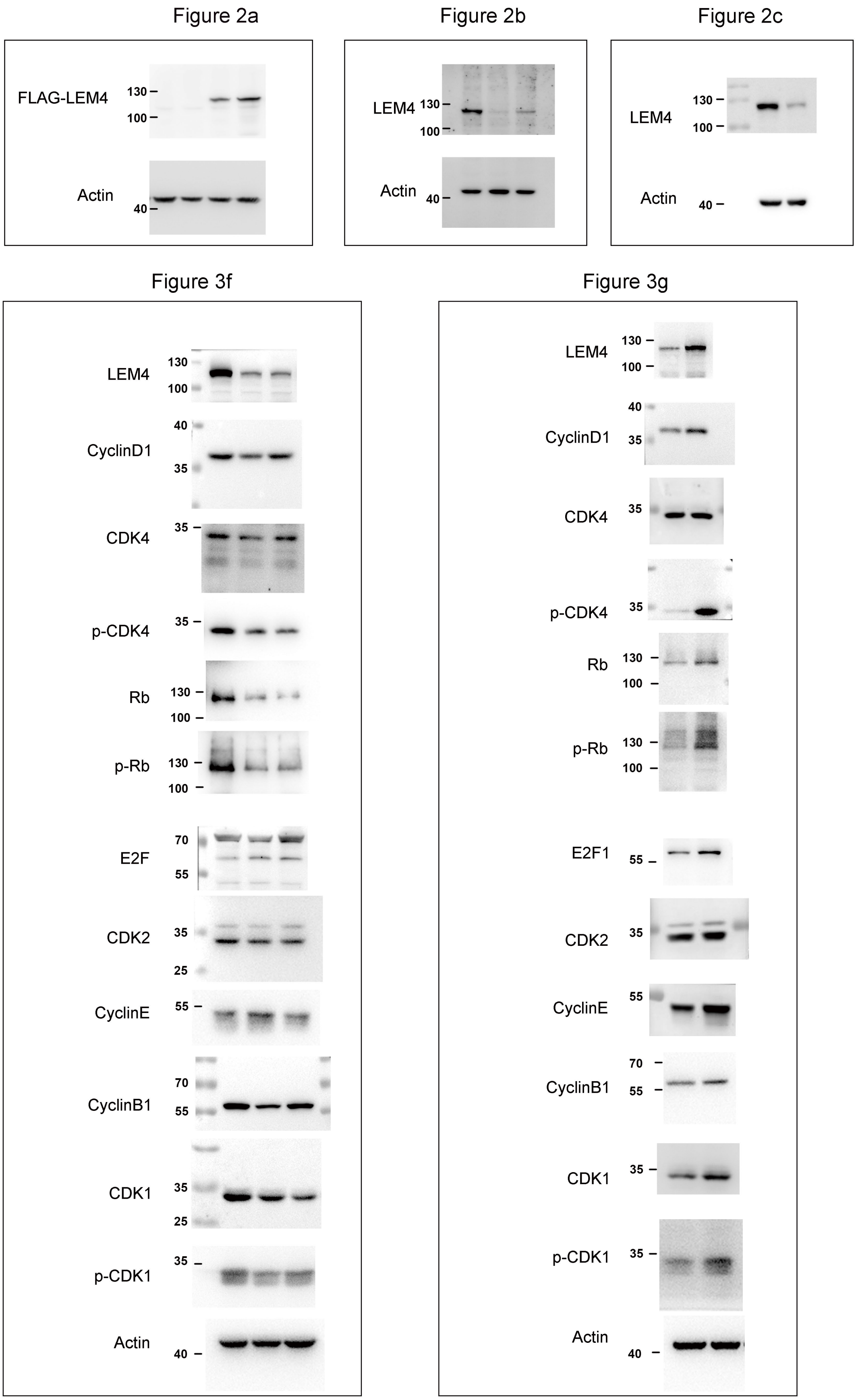

Supplementary Figure 11. Uncropped and unmodified data related to Figure 2 and Figure 3.

Figure 4a

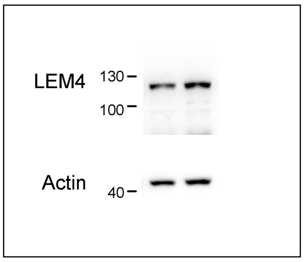

Figure 4c

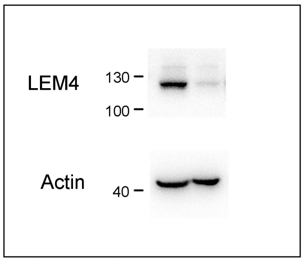

Figure 5b

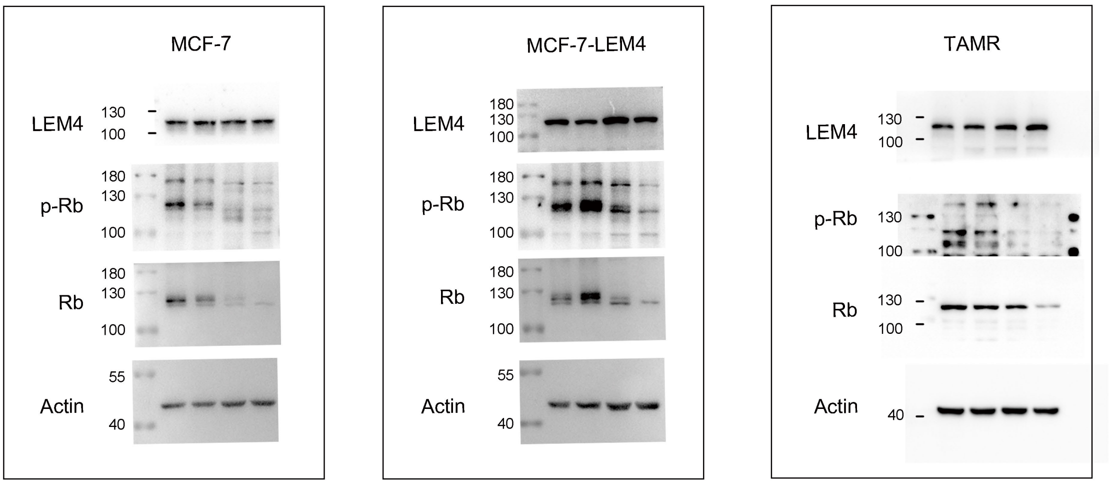

Figure 5e

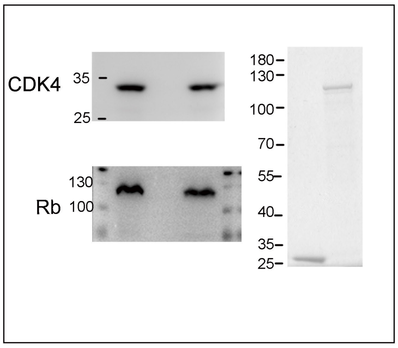

Figure 5f

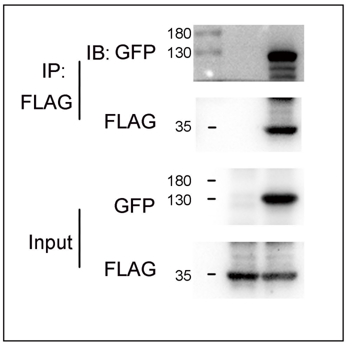

Figure 5g

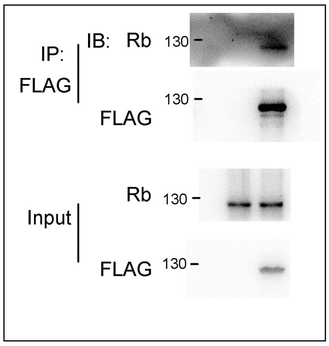

Fig 5h

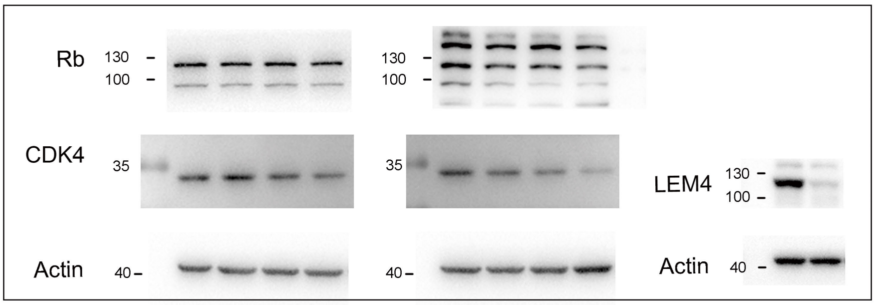

Supplementary Figure 12. Uncropped and unmodified data related to Figure 4 and Figure 5.

Fig 6a

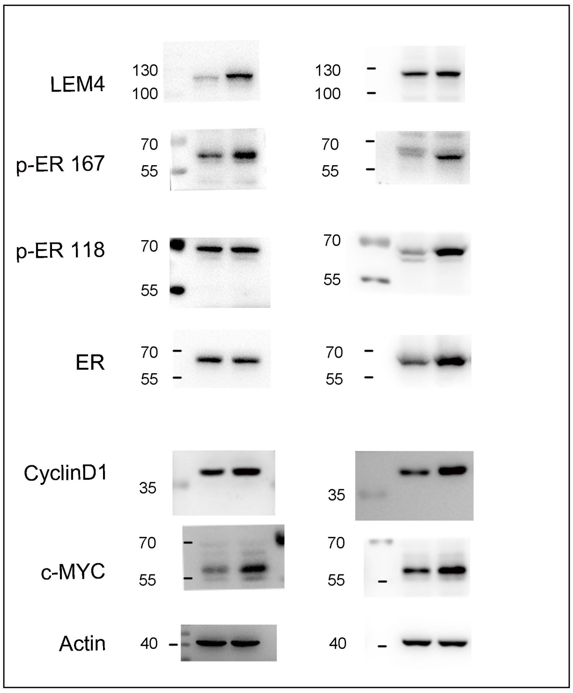

Fig 6b

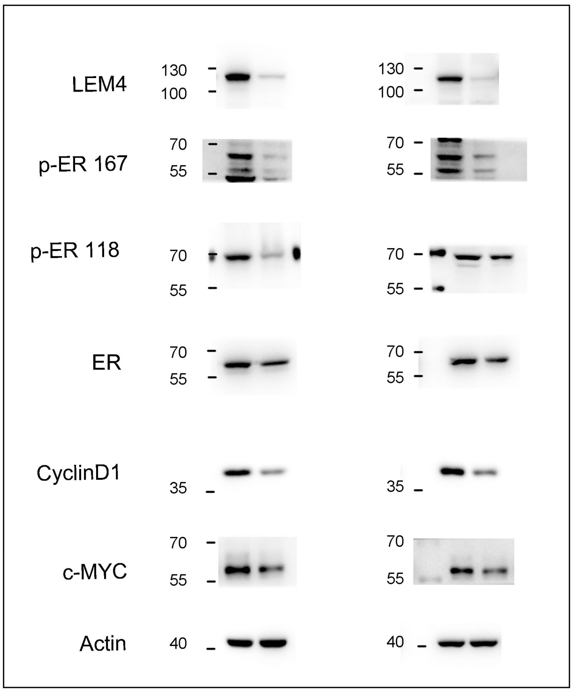

Fig 6c

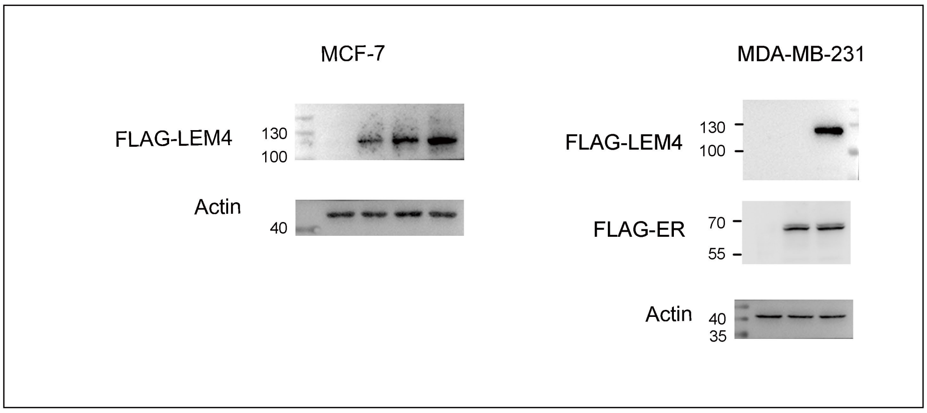

Supplementary Figure 13. Uncropped and unmodified data related to Figure 6.

Figure 7a

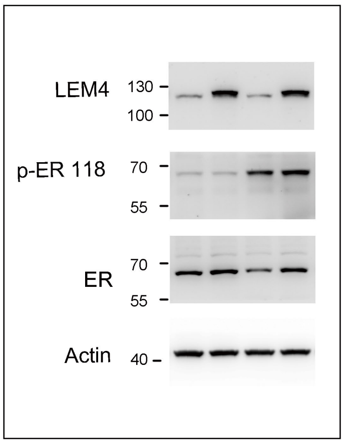

Figure 7b

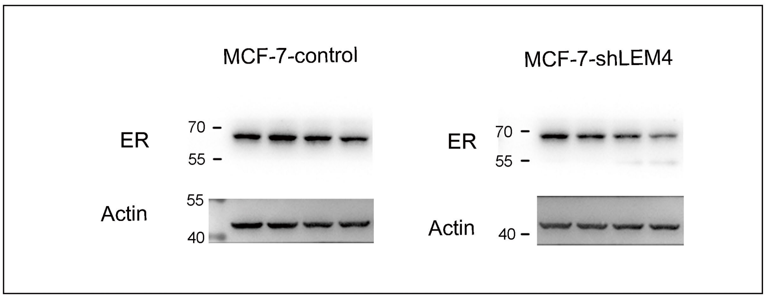

Figure 7c

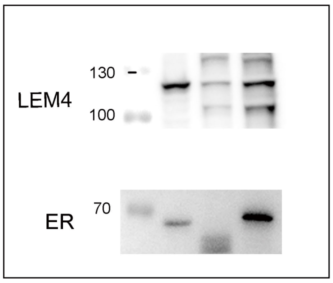

Figure 7d

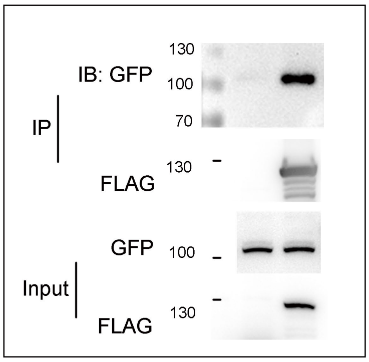

Figure 7e

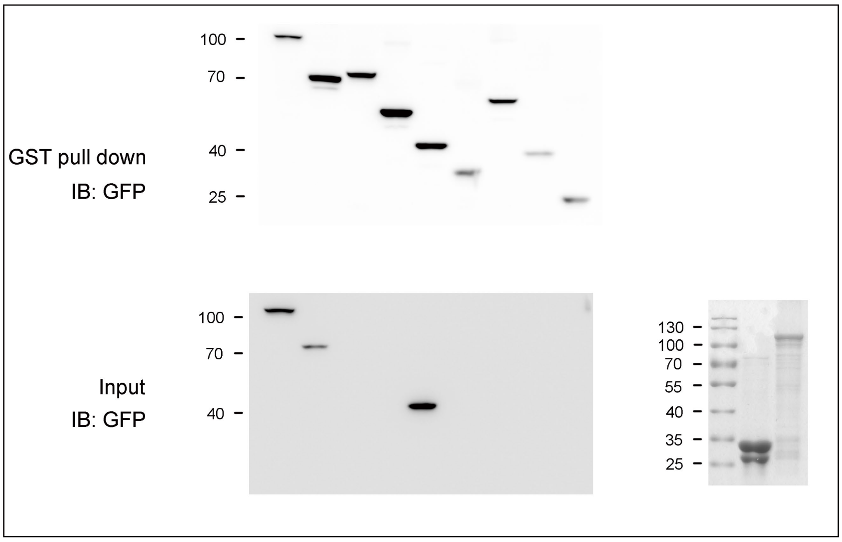

Figure 7g

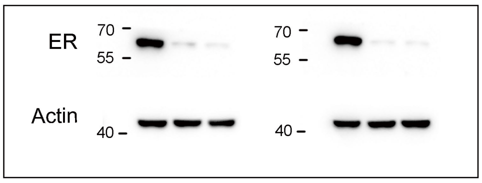

Supplementary Figure 14. Uncropped and unmodified data related to Figure 7.

Figure 8a

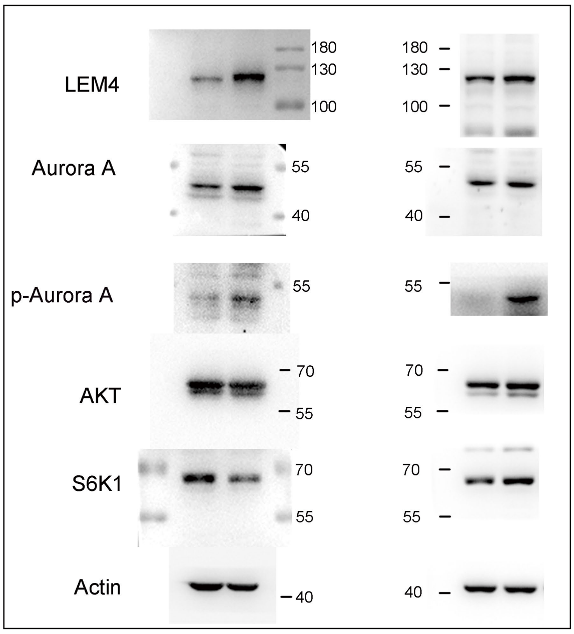

Figure 8b

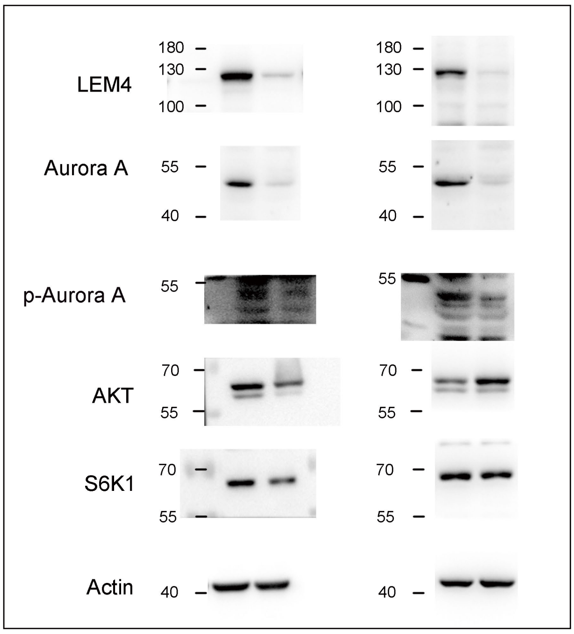

Figure 8c

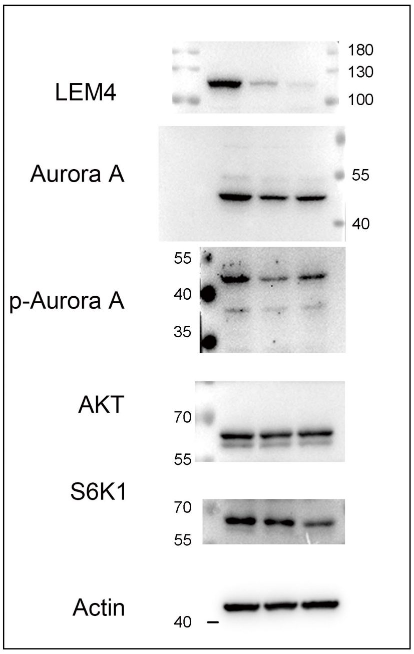

Figure 8d

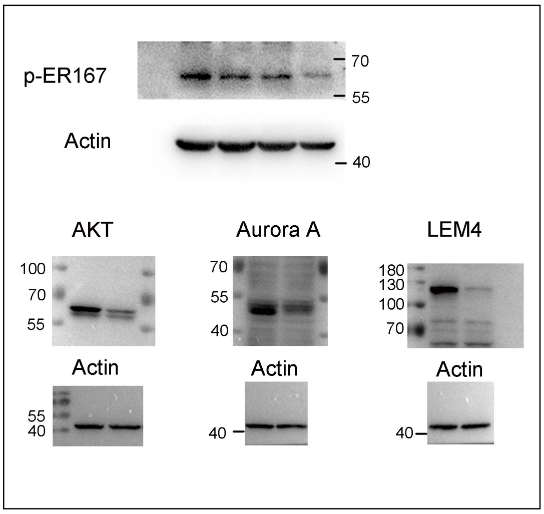

Figure 8e

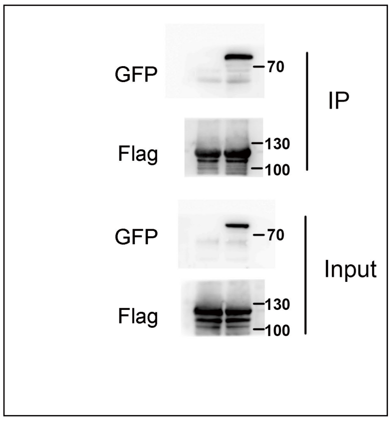

Figure 8f

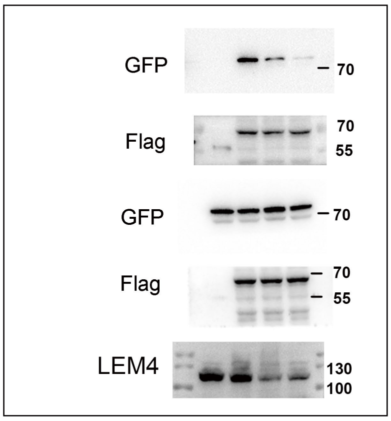

Figure 8g

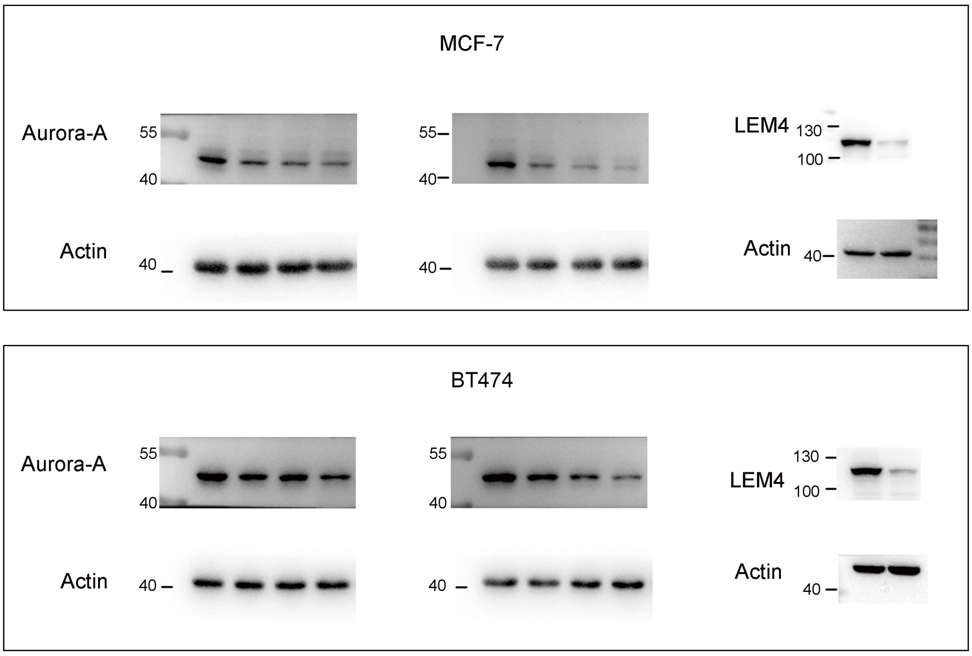

Supplementary Figure 15. Uncropped and unmodified data related to Figure 8.

Supplementary Table 1. The siRNA oligos. Related to Figure 2, 3, 4, 6, 7, 8 and Supplementary Figure 2, 6, 7, 10

Name	Sense sequence	Source
LEM4#1	GAGAAGACGCUGAGAAAUUtt	Invitrogen
LEM4#2	GGUCAUAUGUUUAUUGCUAtt	Invitrogen
AKT	GCUACUUCCUCCUCAAGAAtt	Invitrogen
Aurora-A	AUGCCCUGUCUUACUGUCAtt	Invitrogen
S6K1	AACAGUGGAGGAGAACUAUUUtt	Invitrogen
ERα	UCAUCGCAUUCCUUGCAAAtt	Invitrogen
ERBB2	GCCAACAAAGAAAUCUUAGACGAAGtt	Invitrogen

Supplementary Table 2. The constructs. Related to Figure 5 and Figure 7

Construct	Vector	Forward primer (5'-3')	Reverse primer (5'-3')	Rest Enzyme
GST-LEM4 59-938	pGEX6P1	CGGGATCCGCCTCAG GTGAAATGACAATGG	CGGAATTCCTACAGG GCGGCAAGCTCAGCC	*Bam*H I/*Eco*R I
GFP-ER	pEGFP-C1	CGGGGTACCATGACC ATGACCCTCCAC	CGCGGATCCTCAGAC CGTGGCAGGGAAAC	*Kpn* I/*Bam*H I
GFP-ERN	pEGFP-C1	CGGGGTACCATGACC ATGACCCTCCAC	CGCGGATCCTCACAAC AAGGCACTGACCAT	*Kpn* I/*Bam*H I
GFP-ERC	pEGFP-C1	CGGGGTACCTTGAAA CACAAGCGCCAG	CGCGGATCCTCAGACC GTGGCAGGGAAAC	*Kpn* I/*Bam*H I
GFP-ER1	pEGFP-C1	CGGGGTACCATGACC ATGACCCTCCAC	CGCGGATCCTCACTTG GCAGATTCCATAGC	*Kpn* I/*Bam*H I
GFP-ER2	pEGFP-C1	CGGGGTACCAGGCC AAATTCAGATAATCGAC	CGCGGATCCTCACACT TCACCCCTGCCCTC	*Kpn* I/*Bam*H I
GFP-ER3	pEGFP-C1	CGGGGTACCGGAGGG AGAATGTTGAAAC	CGCGGATCCTCACAACA AGGCACTGACCATC	*Kpn* I/*Bam*H I
GFP-ER4	pEGFP-C1	CGGGGTACCGATGCT GAGCCCCCCAT	CGCGGATCCTCAATGTA GGCGGTGGGCGT	*Kpn* I/*Bam*H I
GFP-ER5	pEGFP-C1	CGGGGTACCCACCAG CGGCTGGCCCA	CGCGGATCCTCAGACC GTGGCAGGGAAAC	*Kpn* I/*Bam*H I

Supplementary Table 3. Primers used for real-time PCR. Related to Figure 3 and Supplementary Figure 4

Name	Forward primer (5'-3')	Reverse primer (5'-3')
LEM4	GGGTTGAATACTGGGAATTTCTGG	TCTCCTGTTTCTTGTTGAGCCTTT
CDK4	CATCGTTCACCGAGATCTGA	CCAACACTCC ACATGTCCAC
CDK6	TGCACAGTGTCACGAACAGA	ACCTCGGAGAAGCTGAAACA
CCND1	GCTGGAGGTCTGCGAGGA	ACAGGAAGCGGTCCAGGTAGT
Rb	ACCTTGAACCTGCTTGTCCTCT	GGCTGAGGCTGCTTGTGTCT
E2F1	CATCCAGCTCATTGCCAAGAAG	GATCCCACCTACGGTCTCCTCA
CCNE1	GCCAGCCTTGGGACAATAATG	CTTGCACGTTGAGTTTGGGT
CDK2	CCAGGAGTTACTTCTATGCCTGA	TTCATCCAGGGGAGGTACAAC
CCNB1	AATAAGGAGGGAGCAGTGCG	GAAGAGCCAGCCTAGCCTCAG
CDK1	CTAGAAAGTGAAGAGGAAGGGGTT	CCATGTACTGACCAGGAGGGAT
Fibronectin	CCATAAAGGGCAACCAAGAG	ACCTCGGTGTTGTAAGGTGG
E-cadherin	TGAAGGTGACAGAGCCTCTGGAT	TGGGTGAATTCGGGCTTGTT
ZEB1	TGCACTGAGTGTGGAAAAGC	TGGTGATGCTGAAAGAGACG
Slug	ATGAGGAATCTGGCTGCTGT	CAGGAGAAAATGCCTTTGGA

Supplementary Table 4. Primers used for ChIP. Related to Figure 6

Name	Forward primer (5'-3')	Reverse primer (5'-3')
TFF1	GACAGAGACGACATGTGGTGAGGTCA	CACCCCGTGAGCCACTGTTGTC
PGR	AATGAGGCTGACATTCTGGGA	GTTGACCTCATTCCAAGGCAG
GREB1	GCTGACCTTGTGGTAGGCAC	CAGGGGCTGACAACTGAAAT
CCND1	CAGTTTGTCTTCCCGGGTTA	TCATCCAGAGCAAACAGCAG
MYC	GCAAACAAATCATGTGTGGGGCT	GGGGAGTCTTGAGCTAATTAAA

Supplementary Table 5. Antibodies related to Figure 1, 2, 3, 4, 5, 6, 7, 8, Supplementary Figure 2, 4, 5, 6, 7, 8, 9 and 10.

Antibody	Source	Dilutions (WB=Western blot, IHC= Immunohistochemistry)
AKT	Cell Signaling Technology (4691S)	1:1,000 for WB
Aurora A	Abcam (ab13824)	1:1,000 for WB
p-Aurora-A (T288)	Abcam (ab18318)	1:500 for WB
CDK1	Abcam (ab18)	1:1,000 for WB
p-CDK1 (Y15)	Abcam (ab133463)	1:1,000 for WB, 1:500 for IHC
CDK2	BD (610145)	1:1,000 for WB
CDK4	Abcam (ab108357)	1:2,000 for WB, 1:500 for IHC
p-CDK4 (T172)	ABclonal (AP0593)	1:1000 for WB, 1:500 for IHC
Cylin B1	Abcam (ab2949)	1:5,000 for WB
Cyclin D1	Abcam (ab134175)	1:10,000 for WB, 1:2,000 for IHC
Cyclin E	BD (551159)	1:500 for WB
Cleaved caspase3/7	Cell Signaling Technology (9661S)	1: 500 for IHC
c-Myc	Cell Signaling Technology (9402)	1:1,000 for WB
ERα	Santa Cruz (SC-543X)	1:1,000 for WB
ERα	Santa Cruz (SC-8002X)	2μg per immunoprecipitation for ChIP
ERα	Abcam (108398)	1:100 for IF
p-ERα (S118)	Abcam (ab32396)	1:1,000 for WB
p-ERα (S167)	Cell Signaling Technology (5587S)	1:500 for WB, 1:500 for IHC
E2F1	Abcam (ab179445)	1:2,000 for WB
E-cadherin	Abcam (ab1416)	1:1000 for WB
FLAG	Sigma (F7425)	1:1,000 for WB, 1:400 for IF
Ki-67	Abcam (ab15580)	1: 500 for IHC
LEM4	Sigma (HPA003602)	1:2,000 for WB, 1:500 for IHC
Rb	Abcam (ab181616)	1:2,000 for WB
p-Rb (S780)	Abcam (ab173289)	1:1,000 for WB, 1:500 for IHC
S6K1	Abcam (ab32529)	1:5,000 for WB
Slug	Cell Signaling Technology (9585s)	1:200 for WB
β-actin	Sigma-Aldrich (A1978)	1:5,000 for WB
